# Unmasking the Difficulty of Listening to Talkers With Masks: lessons from the COVID-19 pandemic

**DOI:** 10.1177/2041669521998393

**Published:** 2021-03-10

**Authors:** Elena Giovanelli, Chiara Valzolgher, Elena Gessa, Michela Todeschini, Francesco Pavani

**Affiliations:** Center for Mind/Brain Sciences - CIMeC, University of Trento, Rovereto, Italy; Center for Mind/Brain Sciences - CIMeC, University of Trento, Rovereto, Italy; Integrative, Multisensory, Perception, Action and Cognition Team - IMPACT, Centre de Recherche en Neuroscience de Lyon, Lyon, France; Center for Mind/Brain Sciences - CIMeC, University of Trento, Rovereto, Italy; Dipartimento di Psicologia e Scienze Cognitive - DiPSCo, University of Trento, Rovereto, Italy; Center for Mind/Brain Sciences - CIMeC, University of Trento, Rovereto, Italy; Integrative, Multisensory, Perception, Action and Cognition Team - IMPACT, Centre de Recherche en Neuroscience de Lyon, Lyon, France; Department of Psychology and Cognitive Sciences, University of Trento, Rovereto, Italy

**Keywords:** speech processing, multisensory, speech in noise, facial masks, COVID-19

## Abstract

Interactions with talkers wearing face masks have become part of our daily routine since the beginning of the COVID-19 pandemic. Using an on-line experiment resembling a video conference, we examined the impact of face masks on speech comprehension. Typical-hearing listeners performed a speech-in-noise task while seeing talkers with visible lips, talkers wearing a surgical mask, or just the name of the talker displayed on screen. The target voice was masked by concurrent distracting talkers. We measured performance, confidence and listening effort scores, as well as meta-cognitive monitoring (the ability to adapt self-judgments to actual performance). Hiding the talkers behind a screen or concealing their lips via a face mask led to lower performance, lower confidence scores, and increased perceived effort. Moreover, meta-cognitive monitoring was worse when listening in these conditions compared with listening to an unmasked talker. These findings have implications on everyday communication for typical-hearing individuals and for hearing-impaired populations.

## Introduction

From the onset of the COVID-19 pandemic, the use of face masks prevented many of us from seeing each other’s lips in everyday interactions. Face masks cover about 60% to 70% of a face, hiding the lower part which is particularly useful for identifying emotional expressions (Blais et al., 2017; Carbon, 2020) and for supporting communication through lip reading (Rosenblum et al., 1996), especially in presence of background noise (Sumby & Pollack, 1954). The few studies that have examined the impact of face masks on communication have reported a mask-induced attenuation of the voice between 2 and 12 dB (Atcherson et al., 2017; Goldin et al., 2020; Mendel et al., 2008) and showed a benefit of transparent masks in hearing-impaired individuals (Atcherson et al., 2017). In five normal hearing adults, Hampton et al. (2020) reported that word-identification performance drops by 30% to 35% when listening with background noise to a talker wearing a mask, compared with a control condition in which the talker’s face was visible. In these previous studies, however, the impact of face masks on speech understanding was rarely examined in common everyday life conditions. Furthermore, the impact of concealing the lips (a visual feature) was not observed separately from the impact of voice distortions (an auditory feature) generated by the mask (i.e., transmission loss: Llamas et al., 2009).

In this study, we aimed to clarify the impact of face masks on speech understanding starting from one of the most common communication modalities imposed by social distancing, namely, video calls. Video conferences can impact on our possibility to exploit audio-visual cues in speech perception and can undermine the listening strategies normally involved in speech understanding (e.g., gazing at the talker), often making speech processing more challenging. In addition, a reduction of visual information about the talker is frequently present in multitalker video calls because talkers can decide to identify themselves through their name only. Finally, face masks can be adopted whenever the talker shares a physical space with others while participating in a video call.

In our study, we developed an on-line experiment that mimicked a real multitalker video call to measure the impact of these different visual conditions on speech comprehension in typical hearing participants. Importantly, while the availability of visual cues changed across conditions (talkers fully visible, talker wearing surgical masks covering the lower part of the face, talkers hidden behind a black screen, and identified only by their names), the audio tracks were always recorded in the same natural conditions (i.e. they were not distorted by the fabric of the face mask, when this visual condition was used). In this way, we dissociated the visual impact of wearing a mask from the auditory impact of wearing a mask.

## Materials and Methods

### Participants

Thirty-six typical-hearing participants (mean age = 26.0 years, *SD* = 4.64, age range = [19–40], six males) took part in the study. All participants gave their informed consent before starting the experiment, which was conducted according to the criteria of the Declaration of Helsinki (1964, amended in 2013) and in accordance with the regulations at the University of Trento. All participants reported typical hearing by self-report (a necessary inclusion criterion).

### Stimuli

**Figure 1.**
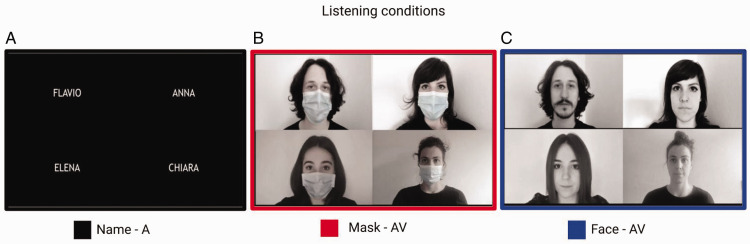
Listening conditions. A: Name condition, auditory only (A), in which talkers were identified only by their names. (B) Face mask condition, audio-visual (AV), in which the lower part of the face of each talker was covered with a surgical mask. (C) Face condition, AV, in which the entire face was visible. An example of the videos used in the study is available at: https://osf.io/8dqbg/

We created an on-line listening-in-noise task that resembled a real video call with multiple talkers (see Figure 1). To prepare the experimental trials, a total number of six talkers (two males and four females) were video-recorded while uttering simple sentences. Each experimental trial was a video with sound (average duration 2.49 seconds, range [1.94–2.93]) that comprised one male talker (target) and one or three female talkers (distractors). Occasional trials with only the male talker were also included, to be sure that participants were paying attention to the task (catch trials). The two male talkers were equally represented in the resulting trials. Importantly, the talkers were either invisible and identified only by their names (Figure 1A), visible from the neck upward with the lower part of the faces covered by surgical masks (Figure 1B) or visible from the neck upward with their faces fully visible (Figure 1C).

All stimuli were prepared during the first Italian lock-down phase (April 2020), when access to the laboratories and gathering were forbidden. Each talker recorded the videos using their own Android smartphone, always in the same room and with similar illumination conditions, using empty white walls as backgrounds. The rooms in which the talkers recorded their videos were domestic furnished rooms (mean volume in m^3^: 38.22; *SD*: 5.38; as indicated by Díaz and Pedrero (2005), mean reverberation time for furnished rooms with volume between 30 and 40 m^3^ is 0.40 seconds; *SD*: 0.08). The distance between the talker and the phone camera was approximately 50 cm. Half of the videos were recorded while the talker wore a surgical mask, whereas the other half was recorded with the face fully visible. Each talker uttered a series of five-words phrases (e.g., “Anna compra dieci matite utili,” which is Italian for “Anna buys ten useful pencils”), adapted from the Italian version of the Matrix test (Puglisi et al., 2015). All phrases were syntactically and grammatically correct but semantically unpredictable to avoid effects of linguistic competence on listening.

Recorded audio and video tracks were treated separately using dedicated editing programs (Audacity 3.2.2 and DaVinci Resolve version 16.2.2.012, respectively). Depending on the smartphone, the audio sampling-rate ranged from 44.1 kHz to 48 kHz. First, each audio and video track was checked for quality (e.g., hesitation or pronunciation mistakes) and if necessary repeated. Next, all audio tracks were equalized in volume using the Contrast Analysis—WCAG 2 tool and saved in MP3 format (audio compression range: 170–210 kbps), before assembling them into the experimental trials. Each experimental trial was the result of two processes: the overlapping of audio tracks for all talkers in the trial and the assembly of the corresponding videos in a 2 × 2 matrix (when the trial comprised four talkers they were arranged as in Figure 1; when the trial comprised two talkers, they were arranged along one of the two diagonals; when the trials comprised a single talker it was placed in either one of the four squares of the matrix). Audio track equalization allowed measuring signal-to-noise-ratio (SNR) for each experimental condition: SNR = 0 dB for one distracting talker trials, SNR = −4.78 for three distracting talkers trials. A silent interval ranging between 850 milliseconds and 1000 milliseconds (50 milliseconds steps) was added to each video to avoid the abrupt onset of the auditory stimuli; when talkers were visible (face and mask conditions), the still image of each talker was presented during this phase.

Crucially, only the audio tracks recorded without a mask were used when assembling the experimental trials. As anticipated, the rationale for this choice was to avoid a confound between the effect of masks on the audio signal and the effect of masks on visual processing of speech. To this aim, each single audio track was paired with each of the three visual conditions. For the face visible condition, the actual video and the corresponding audio track were used; for the name-only condition, the audio track was paired with a black screen with the name of the talker; for the face mask condition, the audio track was paired with the video of the same sentence uttered with the mask, carefully synchronized for onset. When pairing the audio tracks with the corresponding video with face mask, we made sure that the onset and offset of the audio and video tracks matched. To this end, we first compared the audio track obtained in the masked condition with the audio track without mask to detect any difference in synchronization between the two audios; when mismatches were detected, we either rerecord the sentence to fit better the timing of the track without mask (in cases of substantial discrepancies) or adjusted the video speed using the editing tools available in DaVinci Resolve (in cases of minimal discrepancies). Note that the video adjustments were always in the range of few milliseconds and could not be detected by participants.

Ultimately, we obtained three different versions of each sentence, which were used in three different versions of the test to be administered across participants. In this way, the phrases that in the first version were uttered by masked talkers, in the second one, they were uttered by completely visible talkers, and in the third, they were heard while seeing only the talkers’ names.

### Procedure

In the invitation, mail sent to each participant we presented the study and the instructions through a short video. We asked participants to use a computer and to wear earphones. In the first part of the experiment, participants gave their consent to take part in the study and filled in a form reporting their age, sex, and self-assessed hearing abilities. Participants were clearly instructed to pay attention only to the male talker in the videos. They were then asked to write the exact words pronounced by the target talker and then to judge their confidence in what they heard (*Quanto sei sicuro della tua esperienza di ascolto?*, which translates in: *How confident were you about your listening experience?*) and their listening effort (*Quanto e stato difficile ascoltare la voce?*, which translates in: *How difficult was it listening to the [male] voice?*). Both answers were scored from 0 to 9, with 9 indicating *maximal confidence* and *maximal effort*, respectively.

Before starting the test trials, participants performed six sample trials, showing all possible trial types (i.e., the six combinations between number of distracting talkers and listening conditions) to become familiar with the task and to adjust their listening volume to an optimal level. During the task, six target-only videos (2 Male Target × 3 Listening Conditions) were evenly interspersed with test trials to monitor the participants’ performance in the absence of noise (catch trials).

### Analysis

Performance scores were computed as the average count of correct words reported in each trial (from 0 to 5), separately for each experimental condition. Similarly, listening effort and confidence were computed as the average rating expressed in each trial (from 0 to 9, where 0 = *no effort*/*low confidence* and 9 = *great effort*/*high confidence*), separately for each experimental condition. Confidence rating refers to a subjective measure of awareness, that stands for how much people think they have chosen their answers by guessing or not (Dienes, 2008; Dienes & Seth, 2010; Norman et al., 2011; Thiede et al., 2003). Listening effort is the subjective assessment of the perceived difficulty in completing the task (Peelle, 2018). Finally, we extrapolated also a measure of meta-cognitive monitoring, that is, the ability to judge successfully one's own cognitive processes while engaged in a task (Son & Schwartz, 2002), using listening confidence and performance measures in each trial. It is worth noting that metacognitive monitoring was not a task, but a value obtained from the relation between perceived listening confidence and actual performance. Good metacognitive monitoring occurs when, irrespective of performance, the relative confidence judgment reflects its outcome (Dienes, 2008; Dienes & Seth, 2010). When measures are in the same direction (high performance − high confidence or low performance − low confidence), the person shows good monitoring, when the two measures are inconsistent (high performance − low confidence or low performance − high confidence) the participant has poor monitoring abilities. We assessed for each participant the percentage of trials in which the listening confidence judgments produced trial-by-trial matched actual performance (low score/low listening confidence + high score/high listening confidence trials/total number of trials), separately for each listening condition. High performance scores corresponded to three or more correctly reported words, while a low performance score was less than two correctly reported words. High listening confidence score corresponded to confidence ratings of five or more points on the Likert-type scale, while low listening confidence was scored for ratings from 0 to 4.

All data were analyzed implementing linear mixed effects (LME) models in R studio (R Core Team, 2013) using the packages lme4 (Bates et al., 2015), car (Fox & Weisberg, 2020; Weisberg, 2019), and lmerTest (Kuznetsova et al., 2017; see also Ruta et al., 2019). Visual condition and number of talkers were used as fixed categorical predictors, and the intercept of each participant as random effect. First, we examined the model with all main effects and interactions and then reduced the model if appropriate using Bayesian Information Criterion as model fitting criterion. In the results section, we only reported the most appropriate model.

## Results

**Figure 2.**
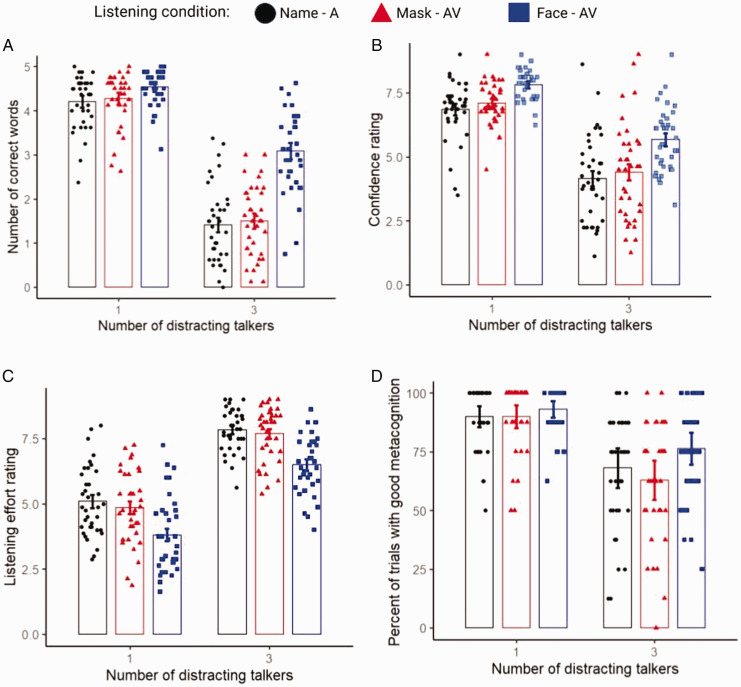
Effects of listening conditions and number of distracting talkers on (A) performance (number of correctly reported words); (B) perceived confidence in the heard words; (C) perceived listening effort; (D) meta-cognitive monitoring, as indexed by the percentage of trials in which confidence judgment and actual performance were concordant.

To study the effect of listening condition (name, mask, and face) and number of distracting talkers (1, 3) on performance, we entered the number of correctly reported words in an LME with listening condition and number of distracting talkers as categorical fixed effects and the intercepts of the participants as random effect. The main effects of distracting talkers’ number and visual condition emerged (see Table 1). As shown in the Figure 2A, seeing the talker’s faces led to better performance compared with seeing faces with masks (*t* = 12.30, *p* < .001) or seeing names (*t* = 13.36, *p* < .001), which instead were comparable (*t* = 1.06, *p* = .53). This listening advantage was especially evident for the noisiest condition.

**Table 1.** Results of the LME Analysis.

	*X* ^2^	*df*	*p*	%Δm*R*^2^_GLMM_
Performance				
Number of distracting talkers	1551.80	1	<.001	39.73
Visual condition	235.26	2	<.001	6.00
Number of Distracting Talkers × Visual Condition	112.65	2	<.001	2.86
Confidence				
Number of distracting talkers	751.31	1	<.001	24.87
Visual condition	137.73	2	<.001	4.54
Listening effort				
Number of distracting talkers	1243.83	1	<.001	32.52
Visual condition	218.18	2	<.001	5.69
Metacognitive monitoring				
Number of distracting talkers	98.65	1	<.001	24.58
Visual condition	9.99	2	.007	2.36

Following previous works (Amenta et al., 2020; Günther & Marelli, 2019), we computed effect size as a percentage increase of marginal *R*^2^ GLMM obtained by adding each parameter to the null model (i.e., the model containing only the random structure) one at a time (Nakagawa & Schielzeth, 2013). Marginal *R*^2^ GLMM values were computed using the MuMIn R package (Barton, 2018). GLMM = generalized linear mixed model.

Similar LME models, applied separately to listening confidence ratings and to listening effort revealed the main effects of distracting talkers’ number and listening condition (see Table 1). Seeing the talker’s face resulted in higher listening confidence, compared with both the mask (*t* = 8.91, *p* < .001) and name conditions (*t* = 11.07, *p* < .001), which instead were comparable (*t* = 2.17, *p* = .08; see Figure 2B). Similarly, seeing the talker’s faces reduced listening effort compared with the mask (*t* = 11.70, *p* < .001) and name conditions (*t* = 13.66, *p* < .001), which were comparable (*t* = 1.96, *p* = 0.12; see Figure 2C).

Finally, when applying the same LME model to the computed metacognitive monitoring measure, both main effects of distracting talkers’ number and listening condition were again found (see Table 1). Participants’ monitoring of their own trial-by-trial performance was better when they saw the talker’s face, compared with when they saw the same person wearing a mask (*t* = 3.089*, p* = .007) suggesting that seeing a talker wearing a mask can hinder meta-cognitive judgments (Figure 2D).

## Discussion

In the present work, we mimicked a real multitalker video call to measure the impact of different visual conditions on speech comprehension in typical hearing participants. Results showed that hiding the talkers behind a black screen or concealing their lips via a face mask led to lower performance and lower listening confidence scores as well as increased listening effort. These differences between listening conditions suggest that the actual audio-visual benefit coming from vision relies on lip reading and demonstrate the impact of face masks on speech comprehension. Understanding a talker wearing a face mask in noise was, in our study, comparable to not seeing him or her at all. Importantly, these findings emerged in a context in which we disentangled the impact of visual information related to wearing a mask from the voice distortions generated by the mask. In this way, our results can be interpreted as the consequences of altering or removing visual information from lip movements in speech processing.

Our visual manipulation also impacted on the ability to successfully judge one’s own cognitive processes while engaged in a task, namely, meta-cognitive monitoring. Face masks reduced meta-cognitive monitoring abilities. In this condition, participants’ listening confidence about their performance was less consistent with their objective performance (e.g., they could be confident about their performance, when in fact their speech comprehension was poor, or vice versa). This result is in line with previous work concerning the effect of face masks on confidence in reading emotions (Carbon, 2020), which found lower confidence and accuracy scores in recognizing expressions displayed by faces wearing surgical masks. This result supports the idea that hiding the lower part of a face undermines the efficacy of a conversation not only linguistically but also from a nonverbal point of view. While this result merits further investigation, it may suggest that when interacting with people wearing a mask, we not only feel less confident about our listening experience overall, but we are also less capable of monitoring whether we understood the message correctly or not. In addition, the confusion they generate on emotional reading of face expressions could further contribute to lowering the efficacy of our everyday life communications, preventing us from reconstructing the emotional tone of a conversation, which could partially contribute to better speech comprehension. This novel result is particularly interesting because compensatory strategies (e.g., asking our conversational partner to speak slower or in a louder voice) are typically triggered by adequate meta-cognitive monitoring of the success of the communication exchange (Boldt & Gilbert, 2019).

In June 2020, the World Health Organization warned about the potential risks and harms of face masks on daily communications. As evidenced by this study, when a talker wears a face mask the listening effort increases, while performance and confidence in what we listen decrease (see also Coniam, 2005; Llamas et al., 2009; Saunders et al., 2020). This could potentially result in stress and misunderstandings during communications, and even lead to risky behaviors, such as pulling down face masks or reducing social distancing while trying to understand each other better. In this study, we intentionally focused on a population of young adults, native speakers of Italian (the language used in the experiment), who reported no hearing difficulties. This is because we reasoned that any effect observed in this sample could only be exacerbated in populations that experience difficulties with language and communication. These populations include hearing children developing their L1, for whom the observation of adults’ mouths can play a key role in an educational context (Spitzer, 2020); hearing children and adults learning a new language (L2); adults and aging people with normal hearing but sensitive to noisy contexts (Tremblay et al., 2015); and obviously all the populations with hearing loss or profound deafness. We believe it is a social priority to extend research on the effects of face masks on communication as well as other aspects of interpersonal perception (such as emotional processing or personal identity identification: Carbon, 2020) to all these populations.

The question arises them of how we can combine safe behavior and effective communication. One approach is to consider the introduction of transparent masks on a large scale. At the moment, they are only used in few medical settings (e.g., in the United Kingdom; Action on Hearing Loss, 2020), but they are gaining increasing attention among the hearing-impaired community (Taylor-Coleman, 2020). Even though this solution may seem the best way to reinstate lip reading into verbal communication, the current generation of transparent masks have several limitations. On the one hand, their materials impact greatly on the high frequencies of the human voice (Corey et al., 2020) affecting consonant perception (Divenyi et al., 2005; Roth et al., 2011). On the other hand, transparent masks are difficult to find because there is only a limited number of producers (Chodosh et al., 2020). Finally, in many countries, these devices are not approved by health authorities.

To conclude, our findings provide a clear example of the audio-visual nature of speech processing, and they emphasize the perceptual and meta-cognitive limitations that result from occluding the face of our conversational partner. From the methodological point of view, our study represents a successful attempt to investigate audio-visual communication using an on-line task and simulating an ordinary listening context, such as the video call with a limited number of talkers. Clearly, when conducting hearing research online, a number of criteria need to be relaxed. It would be important to replicate and extend these observations running similar experimental protocols in a more controlled laboratory context in which individual hearing thresholds are also measured (unlike here). Moreover, it would also be important to increase the number of trials per participant (that said, our linear mixed-effect model approach to the analysis implies that we worked on a dataset of 1728 measures overall). Future experiments should also consider using audio tracks recorded both with and without masks, in order to objectively estimate the actual transmission loss produced by the masks and directly compare the effects of those distortions on speech comprehension. It is clear that such a comparison should necessarily exploit professional audio tools and accurate measures, only obtainable in a laboratory context. Nonetheless, our results agree with a vast literature on the multisensory contributions to speech perception and already provide support to recent petitions that pressured the main video conferencing platforms to offer real-time speech-to-text captioning (Chodosh et al., 2020). Most importantly, our findings indicate that audio-visual communication should be pursued even in the case of the health constraints imposed by a world pandemic. This is necessary for everyone, but especially for those individuals for whom face masks could become a severe obstacle to social inclusion.
